# Principles Determining the Structure of Transition Metals

**DOI:** 10.3390/molecules26175396

**Published:** 2021-09-05

**Authors:** Samuel K. Riddle, Timothy R. Wilson, Malavikha Rajivmoorthy, Mark E. Eberhart

**Affiliations:** 1 Molecular Theory Group, Colorado School of Mines, Golden, CO 80401, USA; skriddle@mines.edu (S.K.R.); twilson@mines.edu (T.R.W.); mrajivmoorthy@mines.edu (M.R.); 2 Department of Chemistry, Materials Science Program, Colorado School of Mines, Golden, CO 80401, USA; 3 Department of Metallurgical and Materials Engineering, Colorado School of Mines, Golden, CO 80401, USA

**Keywords:** quantum theory of atoms in molecules, viral theorem, frontier orbital theory, transition metal structure

## Abstract

For the better part of a century researchers across disciplines have sought to explain the crystallography of the elemental transition metals: hexagonal close packed, body centered cubic, and face centered cubic in a form similar to that used to rationalize the structure of organic molecules and inorganic complexes. Pauling himself tried with limited success to address the origins of transition metal stability. These early investigators were handicapped, however, by incomplete knowledge regarding the structure of metallic electron density. Here, we exploit modern approaches to electron density analysis to first comprehensively describe transition metal electron density. Then, we use topological partitioning and quantum mechanically rigorous treatments of kinetic energy to account for the structure of the density as arising from the interactions between metallic polyhedra. We argue that the crystallography of the early transition metals results from charge transfer from the so called “octahedral” to “tetrahedral cages” while the face centered cubic structure of the late transition metals is a consequence of anti-bonding interactions that increase octahedral hole kinetic energy.

## 1. Introduction

### 1.1. Historical Motivation

One can not help but be struck by how little metallurgical thought has been impacted by the century long advances to the conceptual and theoretical framework of molecular chemistry. Where this framework can be applied, it provides the insights necessary to design molecules and condensed phase systems. Included in the advances contributing to this framework are those derived from valence bond theory [1], one electron and frontier orbital theory [2], conservation of orbital symmetry [3], transition state theory as interpreted within the intuitive approach afforded by the Hammond postulate [4], and the application of catastrophe theory through the formalism of the quantum theory of atoms in molecules (QTAIM) [5]. Though these concepts provide many of the theoretical underpinnings of modern chemistry, they are rarely applied to metallurgical research. We do not believe that the reason for this disparity is that molecular chemistry has little to offer metallurgy. Rather, the two disciplines evolved subject to different selective pressures.

On the one hand, the path that led to the development of modern chemistry arguably began with the 1916 work of Lewis [6], a path to which chemists were fully committed by 1939 with the publication of Pauling’s definitive text, “The Nature of the Chemical Bond” [1]. This text ensconced chemistry as the science of reactivity. The fact that chemical reactions were driven by electron rearrangement had become part of the chemical zeitgeist by 1923 (if not sooner) when Lewis recognized bases and acids as electron pair donors and acceptors [7]. As a result, for chemists of the time, the distribution and response of electrons became the central element of a science devoted to the study of chemical reactions. The actual arrangement of atoms that afforded this response was a byproduct of their investigations. Between 1940 and roughly the mid 1970s, chemistry made tremendous advances through deeper insights into the nature of electron redistribution resulting from a chemical or physical process. (Each of the great advances mentioned in the preceding paragraph occurred in this time period.) During the subsequent years, first principle methods became an increasingly important chemical tool, and though these methods ultimately provided the ability to calculate molecular energies and geometries, their initial utility was derived from the expanded insights they provided toward understanding: (1) how the electrons in a molecules are distributed; (2) how this distribution changes by adding or removing electrons; and (3) the physical basis for these changes.

On the other hand, modern metallurgy is concerned with atomic arrangements and the energy of these arrangements. In the early part of the twentieth century, metallurgical phenomena in which charge rearrangement plays an important role were still not understood. For example, in 1934, Taylor, Orowan, and Polanyi [8,9,10] began individually to zero in on the dislocation as the mechanism of plasticity. Their arguments were based largely on continuum elasticity theory, which is applicable sufficiently far from a dislocation core. However, the energy necessary to displace atoms and create a dislocation (or any defect) fell outside the scope of elasticity theory. Hence, dislocation core energies became a needed parameter in the characterization of slip. Though the resulting elasticity based models were parameter reliant, they provided tremendous metallurgical insight and thus simultaneously made the characterization of atomic arrangements about defects and their corresponding energies a necessary metallurgical goal. Against this backdrop, the utility of maturing first principle methods was judged by their ability to calculate atomic positions and corresponding energies.

This brings us to the current era where, through density functional theory (DFT) [11], it is known that the energy of an atomic system, and all properties that depend on the energy, is determined by the electron density. Chemists take this theory, and the computational tools that it spawned, as an affirmation of their original impulse to study the density and its response. Metallurgists see the density as a stepping stone to calculate atomic positions and their energies.

An interesting question arises as to whether it is possible to combine the many tools and formalisms evolving over the last century to construct models of metallurgical phenomena that lend themselves to a “chemically” based theory of charge rearrangement in metals and alloys. Such a theory would perhaps allow us to apply the tools of molecular chemistry toward a better understanding of phenomena important in the design of metals and alloys. Such an aspiration is beyond the scope of any single study. However, there is a natural starting point. Just as the early advances in chemistry were motivated by attempts to rationalize the arrangement of a molecule’s atoms, in this paper, we investigate the fundamental metallurgical structures, body centered cubic (BCC), face centered cubic (FCC), and hexagonal close packed (HCP). In a very real sense, these structures are the metallurgical equivalent of the sp, $sp^{2}$, and $sp^{3}$ structural building blocks of organic chemistry. In addition, just as the early 20th century chemists did, we will proceed by determining where electrons in each of these structures resides; how this distribution changes with the addition or removal of electrons; and finally we will attempt to develop an intuition as to why these changes occur.

### 1.2. Background

Today, it is a routine matter to calculate ground state crystal structures of fairly complex alloys, and it is a trivial matter to determine the elemental transition metal structures. The results of these calculations are often rationalized in terms of orbital parameters and distributions, e.g., electronic density of states and one electron band energies; see, for example, the excellent reviews provided in references [12,13].

**Figure 1:**
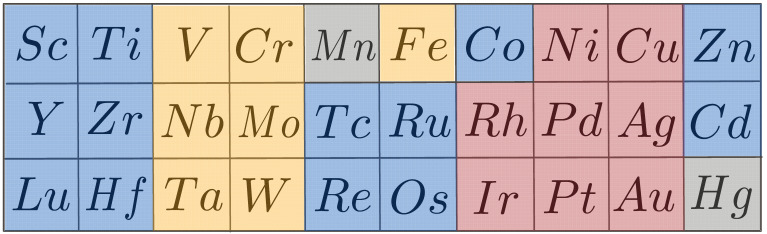
Of the transition metals, only Mn and Hg do not possess one of the three crystallographic structures HCP (**blue**), BCC (**orange**) or FCC (**red**) as ground state structures.

However, there is a rich history associated with attempts by metallurgists and chemists to rationalize the stability of the pure metals as a manifestation of their electron density. In particular, as there are only a few exceptions where the elemental transition metals do not adopt one of three structures BCC, FCC, or HCP, Figure 1, early attempts were directed toward accounting for features of the electronic structure that stabilized each of these structures.

Pauling proposed that interatomic forces in metals could be rationalized from a resonating-valence-bond perspective [14]. Ten years later, Pauling generalized his approach and directly addressed the issue of crystal structure [15]. In the decade following Pauling’s investigations, Altmann et al. [16] employed directed valence bond approaches in an attempt to explain the preferred crystal structure of the non-magnetic transition metals. At nearly the same time, Engel and subsequently Brewer [17], based largely on correlations, suggested that the spherically averaged number of valence *s*-*p*–electrons was the determining factor favoring one metal structure over another. This model, though it survives to this day, was seen as deficient as it assumed *d*-electrons were inconsequential in mediating crystal structure [18]. Along the same lines, other investigators noted a correlation between preferred crystal structures and electrons to atom ratios, e/a [19].

These early attempts to associate crystalline stability with features of the electron density were handicapped by a lack of knowledge regarding the actual electron density associated with a particular crystal structure. With the tools now available, we are no longer so handicapped. Although our tools are modern, our approach has more in common with the methods of Pauling than much of modern computational chemistry.

## 2. Computational Philosophy and Methods

To better motivate our approach, we draw on a delightful article by Roald Hoffman [20] providing a personal assessment concerning the evolution and future of computational chemistry. According to Hoffmann, computational chemistry as a discipline has grown from a devotion to increasingly accurate predictions. Hoffmann articulates the difference between understanding and prediction by arguing that a very accurate computational tool will allow the user to predict a molecule’s properties before it is made. He argues, however, that chemists truly understand the molecule only if they are able to qualitatively predict the outcome of the computation before it is performed. Hoffmann asserts that a computational tool with perfect predictability lacks chemical intuition (understanding); it merely simulates experiments. True understanding is characterized by the ability to rationalize trends.

In this volume we celebrate Linus Pauling’s innumerable contributions to chemistry. As an extremely abbreviated list, consider the concepts that he formulated as a means of fueling chemical intuition: atomic size [21], on which his theory of ionic structure is based [22]; and Pauling electronegativity [23], which among other things allows us to predict substituent effects. Pauling was able to determine an atom’s size by examining lattice constant trends and electronegativity by studying the trends associated with heats of formation. Neither of these parameters is well defined; however, these are exactly the concepts that have proved essential to the qualitative thinking that Hoffmann sees as essential. Hoffmann wonders whether investigations intended to promote qualitative thinking of this kind can survive in an era when exceedingly accurate calculations are possible.

Here, we will take a lesson from Pauling and Hoffmann and analyze the changes to the electron distribution across one row of the transition metal series. Though modern tools provide the capability to analyze these changes in great detail, we will be concerned only with trends. That is, we will be less involved with the absolute magnitude of the changes and more interested in their direction.

Our analysis has three parts. First, we catalogue the electron density topology, that is, the locations of the electron density’s maxima, minima, and saddle points—its critical points (CPs)—for each of the 22 nonmagnetic transition metals in groups 3 through 11 of the periodic table. Next, we investigate how the charge density geometry evolves across the 4*d* transition metal series. In this step, we are interested in determining whether, for example, a charge density minimum becomes deeper or shallower across the series. Finally, we rationalize these observed geometric trends in terms of calculated changes to the kinetic energy distribution, which, in turn, we argue may be understood to arise from fundamental qualitative chemical concepts.

Toward this end, we use several electronic structure methods including the commercial codes BAND [24,25] and VASP [26,27], as well as an older in-house research code employing a linearized augmented Slater-type orbital basis set (LASTO) [28]. Using multiple tools is our way of confirming that our results are not model dependent.

Over some thirty years investigating metallic electron density topology, our group has found that transition metal topology is a robust property, insensitive to variations in lattice constant up to about 10%, code type (e.g., VASP, BAND, or LASTO), k-point mesh, basis set size beyond some reasonable value, and choice of density functional. Thus, here we draw all these calculations together into what we believe is the first comprehensive catalogue of electron density topologies for the nonmagnetic transition metals.

While topology is insensitive to computational parameters, electron density geometry is not. The magnitude of the density at a CP can vary by 5% or more from method to method, different basis sets, or altering the density functional. However, trends persist. Accordingly, we began our calculations of the charge density geometry using LASTO with the Vosko, Wilk, and Nusair local density approximation [29] and employing the zero order regular approximation (ZORA) for relativistic effects [30]. We chose the LASTO code for this step because it was, in part, developed to provide a convenient graphical representation for the analysis of trends. We then used relaxed densities from BAND, correcting for relativistic effects with ZORA, but using the generalized gradient approximation (GGA) Perdew, Burke, and Ernzerhof (PBE) [31] functional to compute the magnitude of the electron density at CPs. Comparison of these values with those found with LASTO confirms that the trends in density across the series are largely model independent, Table A1, Appendix A.

For the final set of results, we used kinetic energy densities and electron counts calculated by BAND for the FCC 4*d* series. These were partitioned using Gradient Bundle Analysis (GBA) [32] into meaningful volumes over which the kinetic energy and electron count is well defined. GBA was performed using the novel and newly developed in-house Bondalyzer add-ons to the Tecplot [33] modeling suite. Each atomic basin employed 20,000 gradient bundles per atom, and no adaptive mesh subdivision.

## 3. Results

### 3.1. Electron Density Topology

A general method of classifying the structure of the electron density—a 3D scalar field—is in terms of its topology, which is fully determined by it extremal points, i.e., the points where the density achieves maximal, minimal, or saddle behavior with respect to neighboring points.

Extremal points are the common critical points (CPs) of Morse Theory [34], and in 3D fields must be either maxima, minima, or one of two kinds of saddle point. These CPs are distinguished by an index giving the number of principal positive curvatures minus the number of principal negative curvatures of the field variable at this point. For example, at a minimum, the electron density curves up (positive) in all directions and obviously also up in the three principal direction; therefore, it is called a +3 CP. A maximum is denoted as a −3 CP because all three curvatures are negative. The two remaining CPs are designated +1 and −1.

CPs have a special relationship to molecular structure [5,35]. For instance, the electron density at an atomic nucleus is always a maximum, −3 CP; and hence it is also called a nuclear CP. Though the existence of non-nuclear maxima was discussed as early as 1955 [36], it was much later that the first such points were discovered in silicon crystals [37,38] and of relevance to this article in the early HCP metals [39]. Such CPs are designated non-nuclear attractors (NNA) or pseudo-atoms.

The other CPs give information about the way maxima are connected. The simplest and perhaps most significant of these connections originates at the −1 CP and terminates at a nuclear CP, which in general lies along a electron density ridge. This path has the topological properties one would expect of a chemical bond. In addition, hence motivated studies showing its presence between nuclei that conventional wisdom assumed to be bound [5,38,40]. Therefore, this ridge is descriptively referred to as a bond path and the accompanying −1 CP as a bond CP. Other types of CPs emerge as chemically meaningful as the connections between nuclei take on additional topological characteristics. A +1 CP is required at the center of ring structures (rings of bond paths), and is designated as a ring CP. Cage structures must enclose a single +3 CP and are given the name cage CPs [41].

Associated with the nuclear and cage CPs are unique energetically well-defined volumes known as Bader atoms and electronic basins, respectively [5,42,43,44]. For crystals having one symmetry unique atom per unit cell, the Bader atom is nothing more than the familiar Wigner–Seitz cell [38]. The shape of this cell is strictly determined by crystallographic symmetry; however, its topology and hence structure is mediated by the types of CPs on its surface.

**Figure 2:**
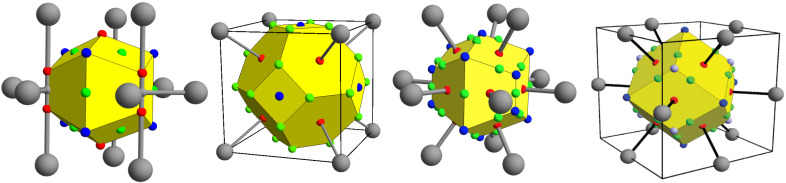
From left to right, the topology of the non-magnetic transition metals of: the early HCP metals of columns 3 and 4; the BCC metals of columns 5 and 6; the late HCP transition metals of columns 7 and 8; and the FCC metals of columns 9, 10, and 11.The yellow polyhedra are the boundaries of the Wigner–Seitz cell about a central atom. The large gray spheres mark the locations of the atoms in the first coordination sphere. Throughout this paper, small grey, red, green, and blue spheres denote pseudo atom, bond, ring, and cage CPs, respectively. Bond paths connecting the first coordination sphere to the central atom are shown as rods.

The electron density topology of crystals having one symmetry unique atom per unit cell is captured by specifying the boundaries of the Wigner–Seitz cell, the locations and types of CPs contained in and on the cell, and the bond paths connecting the nuclear CPs of neighboring cells. Such representations are given in Figure 2 for the four observed ground state topologies characteristic of the 22 non-magnetic transition metals. In regions of flat charge density, there were occasional problems determining topologies, which we attribute to the difficulties involved in interpolating grid data rather than any variation between the methods used. We make note of where such problems occur in the text.

For the nonmagnetic transition metals, the crystallographic HCP structure is characterized by two topologies [39], one for the early HCP metals in columns 3 and 4 of the periodic table and the other for HCP metals of columns 7 and 8. The early HCP (*e*HCP) electron density is absent atom–atom connections; rather, all bond paths pass through pseudo-atom CPs. This pseudo-atom is bound to five early transition metal atoms, requiring bond critical points at the center of HCP tetrahedral hole and cage CPs in the octahedral holes. The crystallographic BCC structure is characterized by eight bond paths to nearest neighbors, a cage CP at the center of the BCC octahedral hole, and a ring CP at the center of the BCC tetrahedral hole. The late HCP (*l*HCP) charge density, as well as the FCC electron density, is as typically conveyed in ball and stick models—each atom is connected to its twelve nearest neighbors via bond paths that produce tetrahedral and octahedral cage CPs in the respective crystallographic holes.

**Figure 3:**
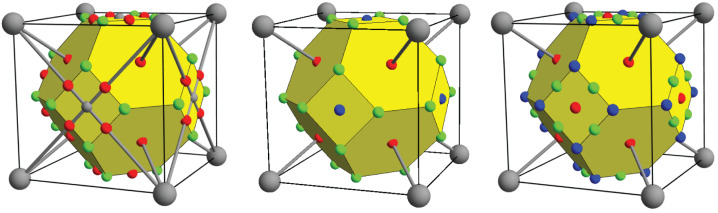
For clarity, all the critical points and bond paths of each topology may not be shown. From left to right, the topologies of the nonmagnetic early HCP, BCC, and late HCP and FCC metals forced the BCC crystallographic structure.

A striking characteristic of these topologies is that, while the crystallographic octahedral hole of all structures hosts a cage CP, the character of the CP at the center of the crystallographic tetrahedral holes depends on the structure—appearing as a bond (for *e*HCP), ring (for BCC), or cage CP (for *l*HCP and FCC). This observation invites the question as to whether this topological variation is due to the atoms involved or simply a response to the crystallographic structure. This question may be enlightened by forcing all the non-magnetic transition metals into a common crystallographic structure—BCC, FCC, or HCP—to determine if the resultant topology of these “forced” structures is the same for all transition metals. Accordingly, the electron density was determined for all of the nonmagnetic transition metals constrained to each of the BCC, FCC, and HCP crystallographic structures. The resultant topologies for the forced BCC structures are represented in Figure 3.

**Figure 4:**
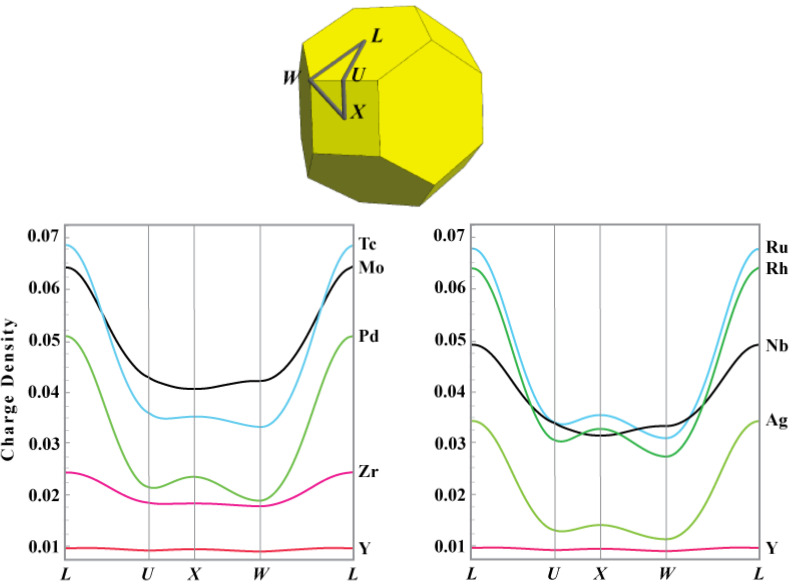
The electron density in AU using the LASTO software package for the 4*d* transition metals around the *L–U–X–W–L* loop. Only for Mo and Nb (representative of the non-magnetic BCC metals) is the electron density at the *W* point other than a minimum around this circuit. The graphs were divided into two sets to enhance readability. Lattice constants provided in Table A2.

Only one forced BCC topology is observed for all the *e*HCP metals (columns 3 and 4) though, with electron density nearly flat around several sites (see Figure 4), topological identification is difficult. Nonetheless, it appears that, for these metals, there is a pseudo-atom CP at the center of the octahedral hole. Bond paths connecting second and third neighbors intersect at these pseudo-atoms. The crystallographic tetrahedral holes now host ring CPs at their center and bond CPs along their edges. The cage CPs of this topology are internal to the Wigner–Seitz cell and are not shown. The BCC metals (columns 5 and 6) obviously possess only the BCC electron density topology, with ring CPs at the tetrahedral holes. When there is forced BCC, both the *l*HCP and FCC transition metals (columns 7–11) share the same topology, with a bond CP in the center of the octahedral hole, indicative of second neighbor bond paths, which, along with the first neighbor bond paths, requires cage CPs at the center of the tetrahedral holes.

The forced BCC topologies are energetically unstable, confirmed by the fact that the metals normally possessing either the *l*HCP or FCC topologies are not observed in the BCC structure even at elevated temperatures. In the case of the *e*HCP metals characterized by a high temperature BCC allotrope (e.g., Zr, Ti), the BCC structure cannot be sustained at low temperatures and normal pressures even through rapid quenching. We will attempt to shed light on features that indicate a forced topology’s instability when compared with a stable ground state. In order to make the question more computationally tractable in the time allotted for this special issue, we will concentrate on explaining the trends across one transition metal row. The 3*d* metals are excluded due to magnetic effects. Of the remaining rows, the 4*d* metals are the more computationally simple, requiring smaller basis sets and being less sensitive to relativistic effects than their 5*d* counterparts. Thus, we will concentrate on the 4*d* series.

### 3.2. Electron Density Geometry

We begin by providing a representation of the electron density geometry (Electron (charge) density topology is a well known, and arguably abused term in chemical literature. The mathematical field of topology refers to the connectivity of a space, while geometry implies the existence of a metric. In the case of chemical analysis, electron density topology rigorously refers to the connection of critical points by critical paths and surfaces, while electron density geometry implies the ability to compare charge densities with the same topology by evaluating the distances between points, paths, surfaces, or other geometric properties such as curvature, etc.) that will allow us to discern how the electrons added to an atom while moving across a row are distributed through the Wigner–Seitz cell. A convenient approach to facilitate this assessment is to borrow from band theory and construct “electron density bands” by plotting the real space electron density along high symmetry directions. Such plots are shown in Figure 4 for the 4*d* series. The point midway between first neighbor atoms is designated as the *L* point. The center of the BCC octahedral and tetrahedral holes are the *X* and *W* points, respectively, and the electron density is shown around the circuit connecting the *L, X, W, and L* points.

As is clear from topological considerations alone, the *X* point is a minimum around the loop only for those metals for which the ground state structure is BCC. However, the electron density geometry reveals that, as one proceeds across the transition metal series, additional density from added electrons is preferentially accumulated at the *X* point. In fact, the electron density difference between the *X* and *W* points serves as a measure of the “proximity” to a BCC transition. Only for the stable BCC metals is this difference negative. For those metals for which there is a stable high pressure or temperature BCC phase (e.g., Ti and Zr), this difference is positive but small. The difference increases across the series with a smaller positive value for *l*HCP than FCC transition metals.

**Figure 5:**
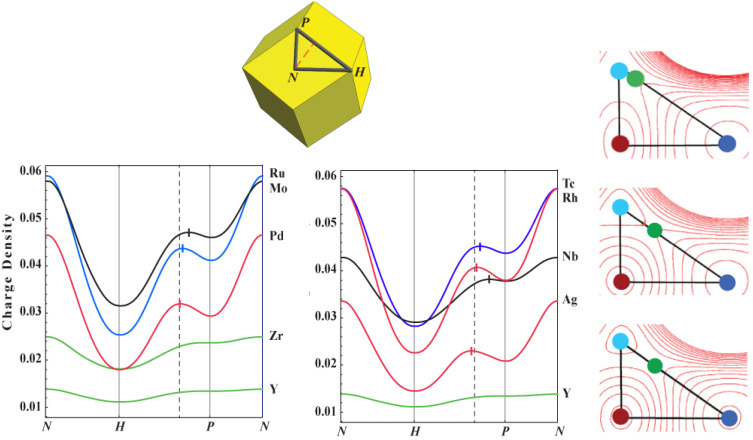
(left) The electron density in AU as generated using the LASTO software package for the 4*d* metals around the *N–H–P–N* loop. The required ring CP between the octahedral and tetrahedral cage CPs is located at the electron density maximum along the *H–P* loop segment and is designated with a cross. The 4*d* metals show identical trends. For the stable FCC metals, this ring CP is located very close to the dashed line designating its ideal position for an FCC transition metal crystal. (The position of the ring CP (green disks) may also be discerned from the contour graphs of the electron density in the appropriate face of the Wigner–Seitz cell for forced FCC: (from top to bottom) V, Cu, and Ir. The graphs were divided into two sets to enhance readability. Lattice constants provided in Table A2.

The pattern of increasingly deeper cage CPs at tetrahedrally coordinated sites while moving from left to right across the transition metal series is repeated for forced FCC structures. Again using the 4*d* series to illustrate, Figure 5 depicts the electron density around a loop on the surface of the FCC Wigner–Seitz cell for the 4*d* metals.

Unlike the forced BCC structures, the character of the CPs at the high symmetry points is the same for all metals, with the possible exception of the *e*HCP metals where the electron density is too flat for conclusive identification. The *N–H–P–N* loop passes through a bond CP, a cage CP at the octahedral hole, another cage CP at the tetrahedral hole, and returns to the bond CP. The character of the CPs around this loop requires a ring CP somewhere along the *P-H* connection, which occurs where the electron density along this line achieves it maximum value.

**Figure 6:**
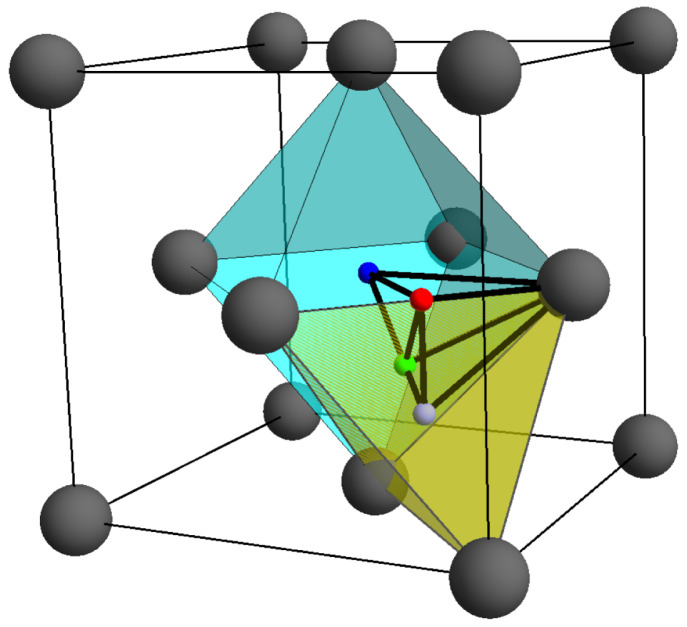
The ideal FCC geometry will be characterized by regular octahedra (turquoise) and tetrahedra (yellow) sharing necessarily flat faces. It is the position of the ring CP that mediates the deviation of an FCC structure from ideality. Transition metals that do not form the FCC structure, when forced, show large deviations from ideally.

Inspection of Figure 5 reveals that, for the early transition metals, this point lies close to (or on) the tetrahedral hole (*P*) and moves toward the octahedral hole (*H*) for transition metals in progressively higher columns of the periodic table. For non-magnetic transition metals with a stable FCC structure, the ring CP is very close to the center point of the flat triangular face shared by a regular octahedron centered on *H* and a regular tetrahedron centered on *P*. This particular FCC geometry we designate as “ideal”, Figure 6. For all transition metals for which the FCC structure is not the stable ground state, the ring CP is located at the center of a bowed triangular face shared by a concave tetrahedron and convex octahedron. This shared face is the ring separating the octahedral and tetrahedral regions of the FCC structure. Though not shown, the same is true for BCC metals forced HCP—the HCP octahedra are convex polyhedra while the tetrahedra are not.

It appears that each of the transition metal crystallographic structures arise in response to preferred electron density topologies and geometries. For example, the presence of a bond CP at the center of the BCC octahedral hole is indicative of an instability, as is a concave tetrahedral cage in an FCC structure. Obviously, these empirically determined relationships between the structure of the electron density and crystallographic structure must have an underlying energetic origin.

## 4. Discussion

We begin our search for these energetic origins by noting that the electron density of an atomic system can be written as a sum over a possibly infinite set Kohn–Sham orbitals, $\phi_{i}$, employing any functional for exchange and correlation, a set that will be referred to here as molecular orbitals, MOs. Then,

(1) $$\rho \left(x\right)=\sum_{i}n_{i}\phi_{i}\left(x\right)\phi_{i}\left(x\right)=\sum_{i}\rho_{i}\left(x\right)$$

where $n_{i}$ is the occupation of orbital *i* and $\rho_{i}\left(x\right)$ is obviously the contribution to the electron density from MO *i*. As has been shown elsewhere [45], the kinetic energy (*T*) of the system at a point *x* can then be expressed as

(2) T(x)=L(x)+G(x)

where

(3) $$L\left(x\right)=-\frac{1}{4}\nabla^{2}\rho \left(x\right)$$

and

(4) $$G\left(x\right)=\frac{1}{8}\sum_{i}\frac{\nabla \rho_{i}\left(x\right)\cdot \nabla \rho_{i}\left(x\right)}{\rho_{i}\left(x\right)}=\frac{1}{2}\sum_{i}n_{i}\nabla \phi_{i}\left(x\right)\cdot \nabla \phi_{i}\left(x\right)$$

The first term of Equation (2) indicates that there is a contribution to the kinetic energy at a point from the Laplacian of the electron density (its total curvature) at this point. The second term arises through the gradient of the MOs from which the electron density is comprised. Orbitals that are more rapidly varying will contribute more to the kinetic energy, which indicates that anti-bonding orbitals will increase T(x) and contribute the greatest increase near interatomic nodes where $\phi_{i}$ varies most rapidly.

Equation (2) is independent of the virial theorem, which, for an atomic system where no forces are acting, requires twice the average kinetic energy ($\bar{T}$) to equal minus the average potential energy ($\bar{V}$), i.e.,

(5) $$2\bar{T}=-\bar{V}.$$

from which it follows:

(6) $$E=\bar{T}+\bar{V}=-\bar{T},$$

leading to the seemingly contradictory conclusion that a system with the larger average kinetic energy will be more stable.

Of note, the virial theorem is not necessarily valid for arbitrarily partitioned subsystems. That is, over individual regions of an arbitrarily partitioned atomic system, the virial theorem need not hold. However, over a class of objects called gradient bundles [46,47] of which nuclear CP centered Wigner–Seitz cells, and cage CP centered electronic basins belong, the virial theorem is strictly obeyed [5]. In addition, the integral of L(x) vanishes over these volumes [5], indicating that the average kinetic energy of a gradient bundle is entirely determined by G(x).

Accordingly, we exploit this fact using our new and unique software [32,48] that allows us to compute and partition the energy of, among other things, chemical bonds and electronic basins. For these calculations, we omitted contributions to kinetic energy due to correlation, $T_{c}$ [49]. In metals, $T_{c}$ was found to be between 0.02 and 0.002% of the total kinetic energy, far smaller than the error introduced from numerical integration, and having no effect on presented trends.

**Figure 7:**
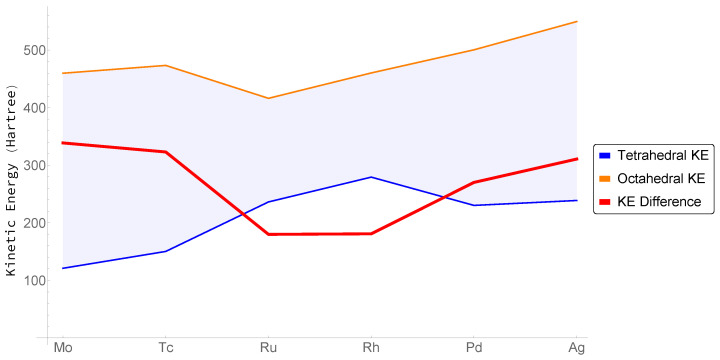
Integrated kinetic energy density (Hartree) in the Tetrahedral (Blue) and Octahedral (Orange) hole, and their difference (Shaded and Red). Lattice constants provided in Table A2.

Thus, we can determine how the energy of the FCC structures shown in Figure 5 is distributed between tetrahedral and octahedral electronic basins, where the energy of the Wigner–Seitz cell is given by summing two tetrahedral hole energies with one octahedral hole energy. This distribution is depicted in Figure 7 for the second row transition metals Mo through Ag. Calculating this distribution for the metals Y, Zr, and Nb was complicated by the flatness of the charged density around high symmetry points P, leading to difficulties determining the topological boundaries of the tetrahedral hole from grid-based data. Nonetheless, these cages are quite small, with significant deviation from the ideal. Their energies must then be small compared to the much larger and expanded octahedral cages.

Regardless, the figure reveals how the kinetic energy and hence total energy is partitioned within the FCC Wigner–Seitz cell for both transition metals that are unstable and stable in this structure. As we move from Mo to Ru, unstable as FCC, the tetrahedral hole preferentially gathers kinetic energy (lowering total energy), while the difference between energy of the octahedral and tetrahedral cages decreases, reaching a minimum at Ru. For the stable FCC structures beyond Ru—Rh, Pd and Ag—it is the octahedral hole that preferentially gathers kinetic energy achieving its maximum value at Ag, while the kinetic energy of the tetrahedral hole achieves its global maximum value at Rh.

We recognize a neutral metallic crystal as being comprised of a number of electron–proton pairs, $N_{e-p}$. For example, we can designate Rh as having an $N_{e-p}$ of 45. Thus, we may define a quantity, $M=\frac{∆E}{∆N_{e-p}}$, which is loosely related to the chemical potential of an electronic basin through the addition of an electron–proton pair i.e., the change in energy of a basin as one steps across a row of the periodic table and approximated by the slope of the connecting segments in Figure 7. We see that, for the stable FCC structures $M_{o}>M_{t}$, where the subscripts *o* and *t* denote the octahedral and tetrahedral electronic basins, receptively, while for those structures that are unstable as FCC, the situation is reversed and $M_{o}<M_{t}$. We note at Rh, where the value of the tetrahedral kinetic energy achieves its maximum value, our simple definition of $M_{t}$ breaks down, as one should properly consider the slopes of the connecting segments both adding and removing an electron. Ideally, one would like to write *M* as a continuous function.

**Figure 8:**
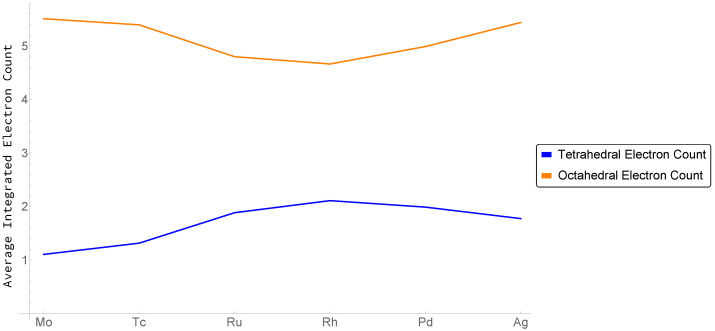
Integrated electron count in the Tetrahedral (Blue) and Octahedral (Orange) hole across the FCC 4*d* series. Lattice constants are provided in Table A2.

Setting aside the complications at Rh (As shown in Table A3, at Rh, we also see an anomalous expansion of the atomic 4*d* and 5*s* valence shell). for the moment, we would expect that, for a chemical-potential-like quantity, electrons would preferentially accumulate in the basin with the larger magnitude of *M*. Inspection of Figure 8 indeed shows that, for elements to the left of Rh, where $M_{o}<M_{t}$, density is gathered by the tetrahedral hole, while to the right of Rh, where $M_{o}>M_{t}$, density accumulates in the octahedral hole.

In this context, recall that crystallographic tetrahedral holes are locations where electron density is either accumulated or depleted. As has been demonstrated from the topologies, proceeding from left to right across the transition metal series, the electron density in regions of tetrahedral coordination varies dramatically, from regions of electron density accumulation (pseudo-atoms) to ones of electron density depletion (cage CPs). Simply, the electron density of the tetrahedral coordinations appears to be indicative of preferred structure.

Let us appeal once more to our chemical intuition, and attempt to motivate this discussion with a one-electron model. We can imagine constructing one-electron trial variational functions by considering the BCC, FCC, and HCP crystallographic structures as constructed from (not necessarily regular) four-atom tetrahedral units. The self-consistent wave functions will then be formed from a linear combination (Block states) of tetrahedral fragment orbitals (FOs) that are in turn derived from atomic-like orbitals centered on tetrahedral vertices. Since, in the solid state, there is approximately one *s*-electron per transition metal (In actuality, the number of s-electrons is basis set and method dependent, but could vary by as much as 10%. The exact value could also effect kinetic energy by the same magnitude, and should be considered as a possible source of error for data presented in Figure 7), the electron density variations across the TM series result primarily from varying occupation of twenty tetrahedral FOs built from *d*-atomic orbitals, each of which can be written as the product of an angular and a radial part.

**Figure 9:**
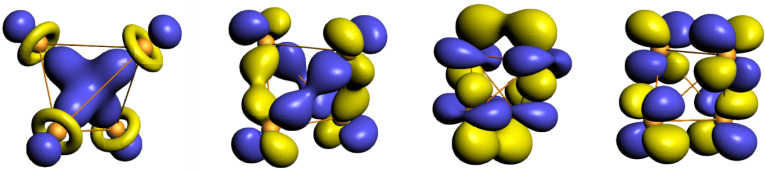
Examples from each class of *d*-derived tetrahedral FOs. From left to right, tetrahedral orbitals that are: a class I FO that is a mixture of σ and π bonding along each tetrahedral edge giving a FO bond order of 6; a class II FO that is σ bonding along four edges and weakly δ anti-bonding along two edges with a bond order of 2; a class III FO that is δ bonding along two edges and π anti-bonding along four edges with a bond order of -2; and a class IV FO that is δ anti-bonding along two edges and π anti-bonding along four edges with a bond order of -6.

These twenty FOs can be placed into four classes distinguished by their net number of bonding interactions (Figure 9), which will correlate with their gradient contributions to the electron density. In the first class are those orbitals characterized by bonding interactions along each of the six tetrahedral edges, possessing a net bond order of six. We note that these orbitals contribute density to the center of the tetrahedron (Figure 9, far left). In the second class are orbitals that are anti-bonding along two opposite tetrahedral edges and bonding along the remaining four, with a net bond order of two. The third class is bonding along two edges and anti-bonding along four edges (bond order -2). These two classes contribute density along some edges and some faces of the tetrahedron (Figure 9, center). Finally, the fourth class is distinguished by orbitals that are anti-bonding along all edges (bond order -6), which has nodes along all edges and passing through all faces (Figure 9, far right).

For all molecules and materials, the first electronic states filled are those that are most bonding while the last filled are the most anti-bonding [50]. Hence, for the transition metals, the first states to be occupied—those at the bottom of the *d*-band—will be formed from a linear combination of FOs drawn from class I; and the last occupied—those at the top of the *d*-band—will be formed from a linear combination of FOs drawn from class IV. Midband states will be formed from a linear combination of class II and III FOs, with a greater contribution from class III FOs as one move up the *d*-band. Quite generally—and entirely consistent with the calculated values of kinetic energy shown in Figure 7—the gradient contributions to the kinetic energy, particularly from the tetrahedrally coordinated regions of the early transition metals, will increase across the series through group 6, smaller for the early transition metals where class I FOs dominate, but substantial for transition metals later in the series where class III and IV FOs begin to fill.

The electron distribution resulting from the filling of these we see played out by inspection of Figure 5, as the tetrahedral hole does not appreciably form until Mo. This is a result of filling class I and II FOs, which, from Figure 9, contributes density to the tetrahedral region. Now, we may turn our attention to the octahedral hole.

**Figure 10:**
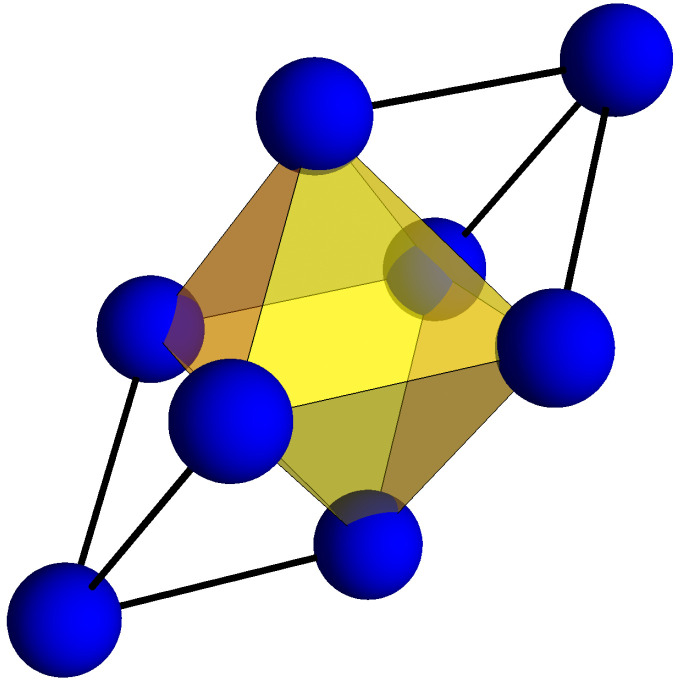
The octahedral coordination may be built from tetrahedra decorating the opposite faces of an octahedron.

To extend our one-electron model to the octahedral hole, consider that an octahedral coordination may be formed from two tetrahedra “decorating” opposite triangular faces of an octahedron (Figure 10). The octahedral electron density may now be thought of as arising from forty FOs, resulting from the bonding (in phase) and antibonding (out of phase) combination of our twenty tetrahedral FOs. The antibonding FOs must possess an interatomic node and hence a steep gradient normal to the nodal plane passing through the octahedral center. As these antibonding orbitals are filled, the contributions to the kinetic energy from the octahedral cage will increase. Simple bonding/antibonding arguments dictate that this filling begins at approximately the middle of the band. In addition, inspection of Figure 7 confirms this process indeed begins with group 9 elements (e.g., Ru) and culminates with the complete filling of the *d*-band.

Returning to Figure 5 shows the octahedral cage deepening faster relative to the tetrahedral cage early in the transition metal series, again consistent with Figure 8 and the fact that $M_{o}<M_{t}$. That is, relative to the superimposed atomic states, electron density is being shifted from the octahedral to tetrahedral hole. As we have seen, early in the transition metal series, so much density is shifted from octahedral to tetrahedral coordinations, the tetrahedral hole does not host a CCP.

By the time we encounter the *l*HCP metals, we have formed both a tetrahedral and octahedral cage, as we fill states that are both predominantly antibonding across the octahedron and tetrahedron. At this point (see Figure 5 and Figure 8), we begin to see a slight decrease in the transfer from the tetrahedral to octahedral cage. However, we also see further effects, which we interpret as a contraction of electron density towards the nucleus. Early transition metals accumulate density near the surface of the Wigner–Seitz cell—the charge density band of Tc is everywhere above that of Mo, which is everywhere above that of Nb, etc. This is what one would expect, as one generally interprets bonding interactions as leading to expansion/polarization of density into the inter-atomic region.

In contrast, though Ag has one more electron than Pd, we see that its electron density band is everywhere below that of Pd, which is everywhere below that of Rh. We note that, across the series, these metals share the same topology, the average electron density is increasing, and the response of system is well modeled by a small number of basis functions (in this case, three Slater-Type orbitals). Based on these facts, we invoke Occam’s Razor, and speculate the observed decrease in density on the Wigner–Seitz cell may be most simply explained as a consequence of radial contraction towards the nucleus. In support of this conjecture, in the absence of a change to the radial distribution due to a bonding/antibonding transition in the middle of the series, we would expect to see a uniform contraction across the series as a result of the increasing effective nuclear charge (see Table A3 for values to this effect in atomic densities). However, contraction of the crystalline electron density across the 4*d* series only becomes apparent with the filling of predominantly antibonding states. This informed speculation regarding the contraction of density implies the average potential energy of the electrons must be decreasing [45,51]. The viral theorem demands there be an offsetting increase in kinetic energy, which in part, comes from filling antibonding orbitals across both the tetrahedral and octahedral cages.

To summarize, we conjecture: early in the 4*d* series, the dominant contributions to lowering of potential (increasing of kinetic energy) comes from charge transfer from the octahedral to the tetrahedral coordinations—yielding structures consistent with that charge transfer—*e*HCP and BCC. Late in the series, stability comes from contraction toward the nucleus to decrease potential and the formation of structures with high kinetic energy tetrahedral and octahedral cages—*l*HCP and FCC. This conjecture is fully testable and is the subject of our current research.

Up to now, our discussion has been restricted to observations which demonstrate clear differences in behavior of the transition metals early and late in the series. However, let us push the envelope, and in the spirit of Pauling conjecture on the origins of stability for *e*HCP, BCC, *l*HCP, and FCC structures.

Consider then an FCC to BCC transformation where one state is metastable and the other is stable. Hence, we can take the total energy as $E=\frac{-T}{V}$ at the endpoints of the transformation. In addition, we ask, “What lowers the kinetic energy through this transition?” Such a transition will manifest through a lengthening of two opposite edges of a tetrahedron and simultaneously shortening of the remaining four edges. The contributions to G(x) from MOs that include contributions from class II FOs that are anti-bonding along the two lengthening edges and bonding along the shortening edges (Figure 9) will diminish through this distortion, lessening the depth of the tetrahedral cage point. If the cage is not too deep, the tetrahedral coordination will be transformed from a source to a sink for electron redistribution. Much as in the case of the *e*HCP structure, charge is redistributed from the octahedral cage to the tetrahedral coordination—in this case to its ring and bond CPs. Diminished charge at the octahedral cage CP and elevated charge in the bond and ring CPs produces a more rapidly varying electron density across the BCC octahedral electron basin and hence increases its kinetic energy.

Contrast this behavior to that of an FCC metal forced BCC. Where the stable BCC topology hosts a cage CP at the octahedral center, the FCC forced BCC topology hosts a shallow bond-CP. Simple analysis will convince the reader that the charge density in this case is more rapidly varying for the stable FCC than forced BCC topology, which would be consistent with an increase in G(x).

The factors stabilizing *l*HCP over FCC are more subtle than those driving the BCC structure. Still the mechanism is consistent with the overall pattern of charge transfer from octahedral to tetrahedral coordinations. Unlike the BCC structure, the magnitude of the charge redistribution is insufficient to transform the tetrahedral cage CP.

**Figure 11:**
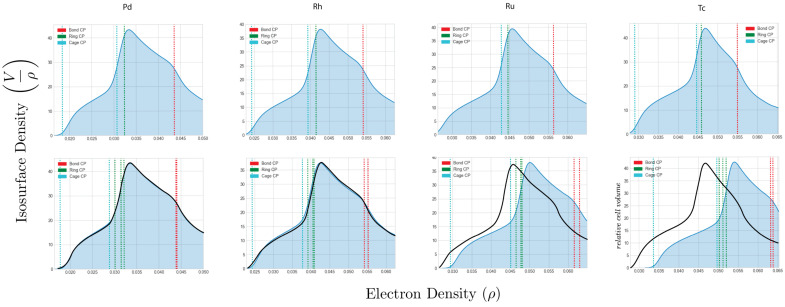
In normalized units the relative fraction of the Wigner–Seitz cell occupied by a given density, i.e., the cell isosurface density, accompanying an FCC to HCP transformation. The top row shows the electron density distribution of the FCC structure and the second row is the distribution of the HCP structure. The normally FCC metals Rh and Pd experience very little charge redistribution through the transformation. For the normally HCP metals, however, charge is observed to be transferred to the bonds and rings of the shared tetrahedral–octahedral faces, making the electron density in the octahedral cage steeper and hence increasing its kinetic energy. Isosurface density may be regarded analogous to a real space DOS partitioning, created by counting the voxels within the Wigner–Seitz cell with a given value of density. The small tails which lie at densities below the cage CPs are fitting artifacts.

The magnitude of the charge redistribution can be inferred from Figure 11, which show the isosurface density through the Wigner–Seitz cell. To briefly explain, the Wigner–Seitz cell was broken down into more than 20,000 voxels. The number of voxels of a given density were then plotted as a histogram, and smoothed to produce Figure 11. These plots depict the change to the density distribution for Pd, Rh, Ru, and Tc when transformed from FCC to HCP. For consistency, we assumed an ideal $\frac{c}{a}$. Of particular note, the electron density distribution changes little when the normally FCC metals (Rh and Pd) are forced HCP. On the other hand, there is a substantive change in the isosurface density of the HCP metals (Tc and Ru) when there is forced FCC. Electron density that was deep in the octahedral cage is shifted to the tetrahedral cage and particularly into the rings and bond paths forming their edges and faces. The density becomes steeper in the octahedral cage particularly in the direction of the *c* lattice vector. Apparently, the lower symmetry about the tetrahedral and octahedral cages of HCP compared to FCC allows the charge redistribution. In the absence of charge redistribution, the FCC structure is preferred simply because the strong electron electron repulsion is minimized by vertex sharing and hence maximizing the distance between the electron rich faces and bonds of the tetrahedral cages.

The charge rearrangement attendant with the transformations of FCC to BCC and *l*HCP are consistent with those of Jahn–Teller transformations. While the exact details of these transformations can be more easily extracted from an orbital perspective (see [52]) at the moment, other than a “not too deep tetrahedral cage”, it is not possible to pinpoint where in the electron density the incipient orbital instability lies.

## 5. Conclusions

We have employed the conceptual and theoretical framework of molecular chemistry in an attempt to rationalize the HCP, BCC, and FCC crystallographic structures of the nonmagnetic elemental transition metals. Specifically, we have drawn on concepts from the quantum theory of atoms in molecules, the molecular virial theorem, and frontier orbital theory to develop a chemically based conceptual understanding of the evolution of electron density topology and geometry as one proceeds from left to right across the 4*d* transition metal series. As a marked departure from conventional metallurgical approaches, where structure is described as the packing of local tetrahedral and octahedral atomic coordinations, we take a broader look at structure by describing it as arising from the packing of topological units characterized by electron density minima at their center.

While the electron density and metallurgical approach lead superficially to the same structural representation for the late transition metals, the electron density approach allows us to go beyond mere energies, and talk about structural changes in terms of charge redistribution between regions. In particular, the structure of the electron density of the early transition metals may be seen to be a consequence of bonding interactions between tetrahedral coordinations. On the other hand, the structure of the late transition metals results from antibonding interactions between the same units. The crystallographic structures between the early and late transition metals can then be rationalized as resulting from mixed bonding/antibonding character, where the preferred structures are those for which the interatomic distance in antibonding directions is lengthened.

Though here we investigated only the nonmagnetic transition metals, the approach is sufficiently general and is applicable to more complex alloys. The challenge is identifying the relevant topological units and then cataloging the possible interactions between these units. As an example, we previously brought this methodology to bear in a study of the structure of metallic glasses [53]. Though complex and confronted with difficulties, the approach allows for a more direct application of chemical formalism. Perhaps the chemical insights that are forthcoming will prove useful in the ongoing effort to design metals and alloys.

## Appendix A

### Appendix A.1. Density Values at Critical Points

**Table A1:** Electron density values (a.u.) at critical points for selected FCC structures from LASTO and BAND results, as referenced in Figure 5. For comparison purposes, BAND results shown here were performed at the LASTO lattice constants, Table A2.

	${\mathrm{CCP}}_{\mathrm{tet}}$	${\mathrm{CCP}}_{\mathrm{oct}}$	RCP	BCP
$Y_{LASTO}$	0.014013	0.013987	0.013895	0.011637
$Y_{BAND}$	0.013767	0.013404	0.0130396	0.010890
$Zr_{LASTO}$	0.025162	0.024378	0.023987	0.018978
$Zr_{BAND}$	0.026599	0.023539	0.023494	0.021422
$Nb_{LASTO}$	0.042969	0.038950	0.038749	0.029567
$Nb_{BAND}$	0.042866	0.041412	0.041045	0.029485
$Mo_{LASTO}$	0.057502	0.048310	0.047882	0.031852
$Mo_{BAND}$	0.056173	0.049881	0.049109	0.032564
$Tc_{LASTO}$	0.057922	0.045897	0.043671	0.028146
$Tc_{BAND}$	0.055938	0.046348	0.045155	0.029412
$Ru_{LASTO}$	0.059638	0.044017	0.041563	0.025472
$Ru_{BAND}$	0.060011	0.042811	0.039057	0.026759
$Rh_{LASTO}$	0.055926	0.041358	0.038629	0.022905
$Rh_{BAND}$	0.053723	0.0399061	0.037307	0.023937
$Pd_{LASTO}$	0.046595	0.032797	0.029381	0.018641
$Pd_{BAND}$	0.043839	0.031556	0.029328	0.018039
$Ag_{LASTO}$	0.020804	0.014512	0.021231	0.033002
$Ag_{BAND}$	0.021419	0.013695	0.022971	0.031606

### Appendix A.2. Calculation Lattice Constants

Slater-type orbital basis used in LASTO results (Figure 4 and Figure 5) were optimized to provide the best match with experimental lattice constants when possible. BAND results (Figure 7 and Figure 8) employed geometry optimized lattices.

**Table A2:** Lattice constants in Angstrom.

	Y	Zr	Nb	Mo	Tc	Ru	Rh	Pd	Ag
$LASTO_{BCC}$	4.20	3.73	3.28	3.13	3.16	3.11	3.10	3.18	3.33
$LASTO_{FCC}$	5.14	4.57	4.02	3.83	3.87	3.80	3.89	3.89	4.08
$BAND_{FCC}$	5.05	4.53	4.22	4.00	3.89	3.82	3.84	3.95	4.15

### Appendix A.3. Average Radial Distance of 4d Electrons

With the exception of Rh, we see a contraction of 4*d* electrons across the series due to increasing effective nuclear charge.

**Table A3:** Average radial distance of 4*d* and 5*s* Electrons (a.u.).

	Y	Zr	Nb	Mo	Tc	Ru	Rh	Pd	Ag
${\langle R\rangle }_{4d}$	2.354	2.297	2.215	2.000	1.723	1.464	1.574	1.356	1.213
${\langle R\rangle }_{5s}$	3.811	3.729	3.730	3.596	3.301	3.251	3.401	3.519	3.034
